# Metagenomic analysis reveals functional potential and storage-driven dynamics of the Kalamata olive microbiome

**DOI:** 10.3389/fmicb.2026.1890405

**Published:** 2026-07-08

**Authors:** Matevz Zlatnar, Rodrigo P. Alves, Gerardo V. Toledo, Wisnu A. Wicaksono, Gabriele Berg

**Affiliations:** 1 Institute of Environmental Biotechnology, Graz University of Technology, Graz, Austria; 2 Solarea Bio Inc., Waltham, MA, United States; 3 Leibniz Institute for Agricultural Engineering and Bioeconomy (ATB), Potsdam, Germany; 4 Institute for Biochemistry and Biology, University of Potsdam, Potsdam, Germany; 5 Department of Colloid Chemistry, Max Planck Institute of Colloids and Interfaces, Potsdam, Germany

**Keywords:** fermented food, food storage, Kalamata olives, lactic acid bacteria, metagenome, microbiome, table olives

## Abstract

**Background:**

Fermented olives are a staple of the Mediterranean diet due to their nutritional value. Despite advances in olive microbiome research, published research on the functional contributions of fermented food-associated microbiota and the impact of storage on these microbial communities remains limited.

**Methods:**

We studied the bacterial communities of ready-to-eat Kalamata olives, stored in glass jars or vacuum-sealed bags at various temperatures (4°C, 8°C and 15°C) for 55-day period. The bacterial abundance, taxonomical composition and functional potential were analyzed by quantitative PCR and amplicon sequencing of 16 rRNA gene, and metagenome sequencing.

**Results:**

The microbiota was dominated by *Lactobacillaceae* (94.6%), a family of lactic acid bacteria (LAB), with dominant genera such as *Pediococcus*, *Lactiplantibacillus* and *Secundilactobacillus*. At the functional level, bacterial genes involved in the biosynthesis of vitamins B1, B2, B5, B7, B9, B12, and vitamin K, as well as short-chain fatty acid metabolism, were observed. Importantly, those functions were not restricted to LAB, underscoring the potential functional contribution of non-LAB taxa to the olive microbiome. Despite conservation, post-fermentation storage, especially the incubation time, temperature, and packaging, influenced the bacterial communities. Lactic acid bacteria were enriched in olives stored at 15°C, whereas non-LAB taxa proliferated more at lower temperatures.

**Conclusion:**

Our study showed that Kalamata olives contain a highly abundant and diverse microbiota that responds to storage practices and carries genes encoding functions that may contribute to the characteristics and quality of the fermented product.

## 1 Introduction

The interconnection between food and the gut microbiome has become increasingly evident. The gut microbiome is widely acknowledged as a significant contributor to human health, playing a key role in nutrient absorption and metabolism, as well as in the regulation of inflammation and immune responses (Donald and Finlay, 2023). A link between human health and the intake of foods rich in microbes, such as plant-based fermented foods, has been proposed (Berg et al., 2025; Marco et al., 2021). For example, in an observational study using the USA-wide health and nutrition examination survey, an additional 100 g per day of foods rich in live microorganisms was associated with improvements in several cardiometabolic risk factors, including lower blood pressure, plasma glucose and insulin, triglyceride, waist circumference, and body mass index (Hill et al., 2023). Recent studies show that food can act as a vector for gut-colonizing microorganisms from the environment (Carlino et al., 2024; Wicaksono et al., 2023) underscoring the significance of diet in shaping gut microbial diversity. These findings support the potential health-promoting role of dietary microorganisms and highlight the importance of addressing current knowledge gaps regarding our food.

Fermented foods have served as a valuable source of microorganisms for centuries. Originally used for preservation, these foods often contain diverse and highly abundant populations of potentially beneficial microorganisms, typically ranging from 10^5^ to 10^9^ microbial cells per milliliter or gram of food, depending on the specific type (Hernández-Velázquez et al., 2024; Rezac et al., 2018). Many food fermentations involve lactic acid bacteria (LAB), which thrive in salty and acidic environments and produce antimicrobial compounds, including lactic acid and bacteriocins, that inhibit most competing microorganisms, allowing LAB to multiply with minimal competition (Perpetuini et al., 2020). In fermented products such as olives and cabbage, several parameters can be adjusted to ensure product safety, but the minimum requirement is the addition of salt at the start of the fermentation, which inhibits the growth of undesirable organisms and favors the growth of desired LAB and yeasts (Martins et al., 2025; Tamang et al., 2020).

Table olives are an ancient and widely consumed fermented product with an annual world consumption of approximately 3,000,000 tons according to the International Olive Council (IOC Statistics Dashboard, 2025). They originate from Mediterranean regions, and the leading producers for the 2024/2025 season are Turkey, Egypt, Spain, Algeria, and Greece (IOC Statistics Dashboard, 2025). Table olives are a staple of the Mediterranean diet, which is linked to a lower risk of chronic diseases and increased longevity (Guasch-Ferré and Willett, 2021). Indeed, olives are of high nutritional value and may promote health due to their richness in monounsaturated fatty acids, vitamin E, fiber, and phenolic compounds (Rocha et al., 2020). Furthermore, olives potentially improve gut health due to their pro- and prebiotic value (Montagano et al., 2024; Rocha et al., 2020). Typically, the olive bacterial community is dominated by *Lactobacillales* (Vaccalluzzo et al., 2020), a well-known group of LAB. A high LAB abundance is crucial for product stability and facilitates the degradation of bitter phenolic compounds, making olives edible (Perpetuini et al., 2020). Kalamata olives are a variety of black olives and are among the most popular Greek types. Although previous studies have characterized the taxonomic composition of the Kalamata olive microbiome (Kamilari et al., 2023; Michailidou et al., 2021), knowledge of its functional potential remains limited. In particular, the metabolic capacity to contribute to nutritional or bioactive properties remains largely unexplored. Moreover, after fermentation and before consumption, olives are typically stored for a period of time, which may influence their microbiome composition. For instance, in the Kalamata olives stored under modified atmosphere conditions, *Pediococcus* and *Lactobacillus* (pre-2020 classification) dominated bacterial communities, but *Pediococcus* decreased drastically after 6 months (Michailidou et al., 2021). Furthermore, inadequate processing or storage conditions can affect food safety and modify microbial communities (Lombardi et al., 2018), and must therefore be understood from a microbiological perspective.

In this study, we employed a comprehensive approach combining shotgun metagenome sequencing, quantitative PCR, and 16S rRNA gene amplicon sequencing to investigate the impact of packaging, temperature, and storage duration on the Kalamata olive microbiome collected in Thessaloniki, Greece. Particular attention was given to LAB, as a major component of the olive microbiome, which remains insufficiently characterized under various storage conditions. The study aims to address two primary research questions: (i) What are the functional potential of the Kalamata olive microbiome? and (ii) How do different storage conditions influence microbial community composition and functional potential? The results presented may contribute to improvements in olive packaging and storage systems.

## 2 Materials and methods

### 2.1 Experimental setup

Kalamata olives were collected in Thessaloniki, Greece, at a farmer’s market on the 12th of October 2023. According to the supplier, the Kalamata olives were spontaneously fermented without the lye treatment. After collection, they were transported and stored in vacuum bags at 4 °C for 6 days until the start of the experiment. Then, baseline samples (*n* = 5 replicates, 5 olives per sample with total weight of approximately 10 g) were collected, pitted and crushed manually, and homogenised in a Stomacher laboratory blender (BagMixer, Interscience, St. Nom, France) with 5 mL sterile NaCl (0.9%) solution for 6 min. For each sample, 1.5 mL of the homogenate was transferred to 1.5 mL Eppendorf tube and treated with PMA to remove the relic DNA (Nocker et al., 2007). Briefly, 1.25 μL of PMA was added to the tube with the homogenate. The tube was inverted several times, incubated in the dark for 10 min, and inverted five times in between. After incubation, the tube was exposed to UV light for 3 min using Chemidoc Universal Hood III (Bio-Rad Laboratories Inc., Berkeley, CA, USA). Samples were then stored at −20 °C until DNA extraction. The remaining olives were used in a factorial design with independent biological replicates (*n* = 3). Briefly, olives (~10 g per sample) were stored either in glass jars or vacuum-sealed bags at 4 °C, 8 °C, and 15 °C. Samples were collected after 9, 30, and 55 days of storage, in accordance with the olive supplier’s recommendation to consume the olives in approximately 2 months. Independent replicate samples were used for each storage condition, duration, and packaging material. All samples were processed using the same procedures as the baseline samples.

### 2.2 DNA extraction

Total DNA was extracted from olive samples using MasterPure™ Complete DNA and RNA Purification Kit (Lucigen, Epicentre; USA) with a modification at the sample lysis step. A total of 0.75 mL of homogenised sample was centrifuged at 4000 × *g* for 5 min. The resulting pellet was resuspended in 500 μL of Tissue and Cell Lysis Solution containing 2 μL of lysozyme solution. The mixture was transferred to a lysing matrix tube containing glass beads (MP Biomedicals, CA, United States) and homogenised using FastPrep Instrument (MP Biomedicals, CA, United States), followed by incubation at 37 °C for 20 min. Then, 2 μL of Proteinase K was added and DNA extraction proceeded according to the manufacturer’s protocol. DNA quantity was measured using Qubit Fluorometer (Invitrogen, Carlsbad, CA, USA). The DNA extracts were frozen at −20 °C until further use.

### 2.3 Quantification of total bacteria and lactic acid bacteria (LAB) using quantitative PCR (qPCR)

The qPCR was performed on QTOWER3 G (Analytik Jena, Jena, Germany). Primer pair 515f (5′-GTG YCA GCM GCC GCG GTA A-3′) and 806r (5′-GGA CTA CHV GGG TWT CTA AT-3′) (Caporaso et al., 2012) with addition of PNA clamps (Lundberg et al., 2013) was used for quantification of total bacteria and primer pair Lact-F (5′-TAT GGT AAT TGT AGC AGT AGG GAA TCT TCC A-3′) and Lact-R (5′-AGT CAG CCA GGG ATT YCA CCG CTA CAC ATG-3′) (Walter et al., 2001) was used for quantification of the main genera of lactic acid bacteria (LAB) namely *Lactobacillus* (pre-2020 classification), *Pediococcus*, *Leuconostoc* and *Weisella*. Those genera are collectively referred to as LAB throughout this manuscript. The qPCR reaction consisted of a total volume of 25 μL, including 1 μL of 10× diluted DNA template, 12.5 μL of KAPA SYBR® FAST qPCR Master Mix (2X) (KAPA Biosystems, USA), 0.62 μL of both primers [10 μM], 9.36 μL H_2_O and for total bacterial quantification, 0.45 μL of mPNA and pPNA oligomers (both 50 μM), for LAB quantification, an additional 0.9 μL H_2_O. Negative control reactions contained PCR-grade water instead of DNA template. To amplify the total bacterial marker gene, the cycling program was as follows: initial denaturing step at 95 °C, 3 min; 39 cycles of 95 °C, 5 s; 78 °C, 5 s; 54 °C, 20 s; 72 °C, 5 s; and a final melting curve. The cycling program for LAB quantification was as follows: an initial denaturing step at 95 °C for 10 min; 45 cycles of 95 °C for 30 s, 60 °C for 30 s, and 72 °C for 30 s; and a final melting curve. qPCR was performed in three technical replicates.

### 2.4 Shotgun metagenome and amplicon library preparation and sequencing

To assess potential microbial functions, we conducted shotgun metagenomic sequencing on baseline samples collected before the storage experiment. Two extracted DNA samples from baseline samples were randomly chosen and sent to Novogene (Cambridge, UK). Novogene conducted DNA library preparations and performed sequencing using an Illumina NovaSeq 6,000 platform, generating 2 × 150 bp paired-end reads.

To evaluate changes in the microbial community during storage, total DNA was subjected to amplicon PCR targeting the V4 region of prokaryotic 16S rRNA genes. Extracted DNA was used for amplicon sequencing library preparation. The V4 region of prokaryotic 16S rRNA gene was amplified using the primer pair 515f (5′-GTG YCA GCM GCC GCG GTA A-3′) and 806r (5′-GGA CTA CHV GGG TWT CTA AT-3′) (Caporaso et al., 2012) with sample-specific barcodes attached to the primer for multiplexing. Peptide nucleic acid (PNA) clamps were added to the PCR reaction mix to prevent amplification of mitochondrial and plastid DNA from the plant host (Lundberg et al., 2013). Twenty-five μL reaction mixture contained 1 μL of 10 x diluted DNA template, 12.5 μL of 2 x KAPA Taq Ready Mix (Kapa Biosystems), 0.62 μL of both primers with barcodes [10 μM], 9.36 μL H_2_O and 0.45 μL of mPNA and pPNA oligomers (both 50 μM). Cycling program was 96 °C, 5 min; 28 cycles of 96 °C, 1 min; 78 °C, 5 s; 54 °C, 1 min; 74 °C, 30 s; and final extension at 74 °C, 10 min.

Additionally, LAB-specific primers, namely Lact-F (5′-TAT GGT AAT TGT AGC AGT AGG GAA TCT TCC A-3′) and Lact-R (5′-AGT CAG CCA GGG ATT YCA CCG CTA CAC ATG-3′), were used to amplify V3 and V4 regions of the LAB 16S rRNA genes (Walter et al., 2001). Each forward and reverse primer included a designated primer pad (TATGGTAATT/AGTCAGCCAG) and linker (GT/GG), as outlined in the protocols of the Earth Microbiome Project to attach barcode sequences to amplicon products in a subsequent PCR step. For the amplification of the LAB 16S locus, 10 μL contained 1 μL of 10 × diluted DNA template, 5 μL of 2 × KAPA Taq Ready Mix (Kapa Biosystems), 0.1 μL of both primers with barcodes [10 μM], 2.6 μL H_2_O and 1.2 μL MgCl_2_. Cycling program was 95 °C, 2 min; 35 cycles of 95 °C, 30 s; 61 °C, 1 min; 72 °C, 30 s; and final extension at 72 °C, 7 min. For multiplexing, sample-specific Golay barcodes were attached to the primer pads in a second PCR. Thirty μL reaction mixture contained 2 μL of PCR amplicon template, 15 μL of 2 × KAPA Taq (Kapa Biosystems) and 1.2 μL of both forward and reverse barcodes [5 μL] and 10.6 μL H_2_O. Cycling program was 96 °C, 5 min; 20 cycles of 95 °C, 30 s; 53 °C, 30 s; 72 °C, 30 s; and final extension at 72 °C, 10 min. PCR targeting both prokaryotic and LAB-specific marker gene regions included negative controls that contained no extracted DNA. All PCR reactions were performed in technical duplicates and combined before the quality control using gel electrophoresis. Afterwards, PCR products were purified using Wizard SV Gel and PCR Clean-Up System (Promega, Madison, WI), pooled together, and sequenced on an Illumina NovaSeq 6,000 (2 × 250 bp paired-end reads) by Novogene GmbH (Munich, Germany). Amplicon and shotgun sequences were deposited at the European Nucleotide Archive (ENA) under the project number PRJEB110067.

### 2.5 Bioinformatics

Prior to analysing the taxonomic and functional diversity of bacterial communities in the olive samples based on shotgun sequencing data, data preprocessing steps were carried out. This included removal of Illumina sequencing adapters and initial quality filtering to exclude low-quality reads with Phred scores below 20, utilising Trimmomatic v0.39 (Bolger et al., 2024) and VSEARCH v2.28.1 (Rognes et al., 2016). Illumina adapter sequences were removed using the UniVec_Core database. For functional gene profiling, metagenomic reads were assembled using Megahit v1.2.9 (Li et al., 2015) in meta-sensitive mode, retaining only contigs longer than 1 kb for further analysis. Open reading frames were predicted using Prodigal v2.6.3 (Hyatt et al., 2010), and duplicate sequences were clustered with mmseq2 to produce a non-redundant gene catalogue at 95% nucleotide identity (−-min-seq-id 0.95) (Steinegger and Söding, 2017).

To identify functionally relevant bacterial genes, the non-redundant genes were annotated using DIAMOND (blast algorithm) in sensitive mode in combination with eggNOG-mapper v2.1.12 (Buchfink et al., 2015; Huerta-Cepas et al., 2017) and the eggNOG v5.0 database (Huerta-Cepas et al., 2019). Only genes assigned to bacteria based on eggNOG taxonomic classification were retained for downstream analyses. Gene abundance profiles were generated by mapping quality-filtered reads back to the non-redundant gene catalogue using BWA v0.7.18 and SAMtools v1.10 (H. Li and Durbin, 2010; H. Li et al., 2009).

Multiple binning algorithms—MaxBin2 v2.2.7 (Wu et al., 2016), MetaBAT2 v2.12.1 (Kang et al., 2019), and CONCOCT v1.1.0 (Alneberg et al., 2014) were employed to assemble MAGs from metagenomic data. MetaBAT2 was run using contigs ≥1 kb and coverage profiles generated from read mapping. MaxBin2 employed abundance estimates derived from mapped read coverage. For CONCOCT, contigs were cut into 10-kb fragments before binning, and coverage tables were generated using mapped read data. The highest quality bins were selected using DASTool v1.1.1 (Sieber et al., 2018). The completeness and contamination levels of MAGs were assessed with CheckM v1.0.13 (Parks et al., 2015). Only medium-quality MAGs, defined as those with more than 50% completeness and less than 10% contamination per MIMAG standards (Bowers et al., 2017), were retained for further analysis. Dereplication of MAGs was performed using dRep v2.2.3 (Olm et al., 2017) to generate a non-redundant set. Taxonomic classification of MAGs was conducted using GTDB-Tk, and phylogenetic relationships were inferred with PhyloPhlAn (Chaumeil et al., 2020). Functional annotations of MAG genes were performed with METABOLIC v4.0 (Zhou et al., 2022). Abundance of each MAG was estimated in terms of RPKM (reads per kilobase per million reads) by mapping reads using CoverM v0.4.0^1^, calculating the number of mapped reads per MAG, normalising by MAG length, and the total number of mapped reads per million in the sample.

For the processing of raw sequencing data, primer and barcode sequences were removed, and demultiplexing was conducted using Cutadapt (Martin, 2011). Demultiplexing was performed using exact barcode matching (error rate = 0, no indels allowed). Subsequent analysis was performed using QIIME 2 (Bolyen et al., 2019). Quality control, the generation of amplicon sequence variants (ASVs), and the removal of chimeric sequences were carried out employing DADA2 (Callahan et al., 2016). Forward and reverse reads were truncated at 160 bp, while no bases were trimmed from the 5′ ends of either read (trunc-len-*f* = 160, trunc-len-r = 160, trim-left-*f* = 0, trim-left-r = 0). Taxonomic classification was performed utilising VSEARCH (Rognes et al., 2016), with reference databases including SILVA v138 (Quast et al., 2013) for bacterial and LAB 16S rRNA genes.

### 2.6 Statistical analysis

All statistical analyses and visualisations were conducted in R v. 4.4.1 (R Core Team, 2024). To determine the impact of packaging, storage time, temperature, as well as their interaction on microbial marker gene copy numbers, a three-way aligned rank transformation analysis of variance (ANOVA) was conducted using the ARTool package (Wobbrock et al., 2011). Microbial community analysis was performed using the R package phylloseq (McMurdie and Holmes, 2013). Before the analysis, reads not assigned to bacteria or *Lactobacillales* were removed from the total bacterial and LAB datasets, respectively. To normalise the data for alpha- and beta-diversity analyses, reads were subsampled to the lowest read count across samples. The total bacterial amplicon dataset was subsampled to 14,652 reads, and the LAB-specific dataset was rarefied to 1,424 reads. These sequencing depths were sufficient to capture most of the microbial diversity present in the samples, as indicated by the rarefaction curves. Six samples were removed from the LAB dataset due to low read counts (428–1,186 read counts) after subsampling. For alpha diversity, the Shannon diversity index (H′) was calculated, and ANOVA was used to compare the effects of packaging, storage time and temperature. For beta-diversity analysis, Permutational Multivariate Analysis of variance (PERMANOVA) and beta dispersion on non-metric Bray–Curtis dissimilarity matrices were performed using the adonis2 and betadisper functions from the vegan package (Oksanen et al., 2007). Differentially abundant taxa were identified by LEfSe (Segata et al., 2011) implemented in microbiomeMarker (Cao et al., 2022) with CPM (counts per million) transformed data.

## 3 Results

### 3.1 Kalamata bacterial community composition and genetic potential

Amplicon analysis revealed a diverse microbiome associated with table olives. Using a universal bacterial primer, we detected 135 unique bacterial ASVs belonging to 71 genera. When targeting LAB specifically, 54 unique ASVs belonging to 7 different genera within the order *Lactobacillales* were identified. Notably, LAB were the most abundant group in the total bacterial communities of olive samples and dominated the olive microbiome: 94.6% of reads from universal bacterial primers were assigned to *Lactobacillaceae*, whereas *Bacillaceae* and *Staphylococcaceae* accounted for only 1.7 and 1.0% of reads, respectively. The remaining bacterial families, including *Enterobacteriaceae*, *Pseudomonadaceae*, *Shewanellaceae*, and others, were detected at low relative abundances, each accounting for less than 1.0% on average. The analysis of the reads obtained using LAB-specific primer pair revealed four highly abundant species: *Pediococcus damnosus* (51%), *Lactiplantibacillus plantarum* (19%), *Secundilactobacillus paracollinoides* (12%), and *Pediococcus ethanolidurans* (4%). The remaining 14% were classified as unclassified *Lactobacillaceae* species and other taxa.

**Figure 1:**
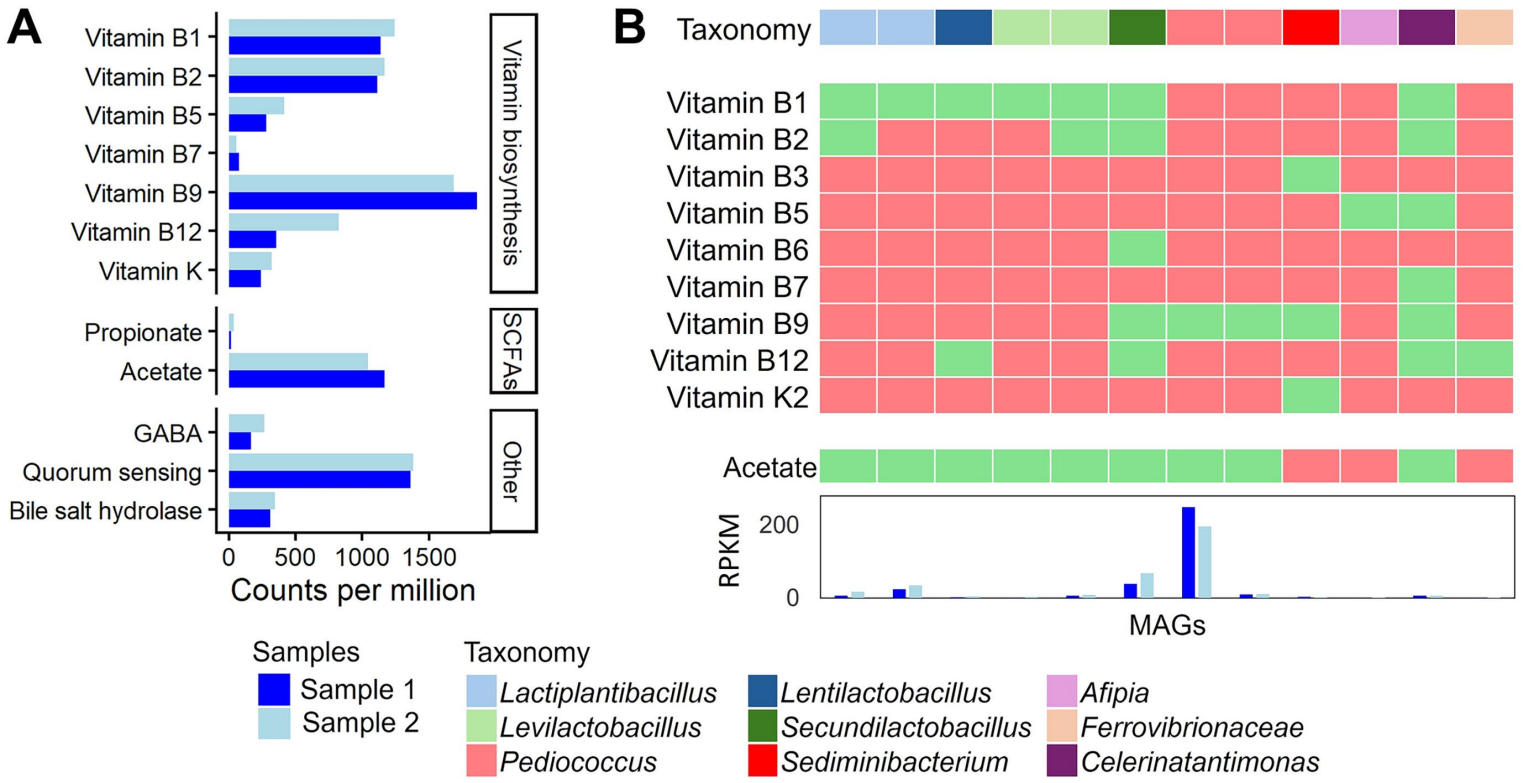
Functional potential of the olive microbiota based on short-read analysis and functional annotation of metagenome-assembled genomes (MAGs). The figure shows the abundance of key genes involved in the biosynthesis of health-relevant compounds in counts per million of bacterial reads. Different shades of blue represent individual samples **(A)**. Biosynthetic pathway module presence (green) or absence (red) for selected functions in metagenome-assembled genomes (MAGs) constructed from the olive metagenome. The visualization includes MAG taxonomy on the genus level and abundance based on reads per kilobase per million reads (RPKM) in the two samples marked by different shades of blue **(B)**.

We utilized metagenomic dataset to investigate bacterial genes associated with human health. Sequencing generated 141,850,393 quality-filtered reads, of which 122,111,278 were mapped to bacterial genes and used for downstream taxonomic analyses. Our analysis revealed a high abundance of genes involved in the biosynthesis of vitamins, short-chain fatty acids, bile salts degradation and quorum sensing. Notably, genes involved in tetrahydrofolate (vitamin B9) biosynthesis - *folE, folB, folK, folP, folC, folA, pabA, pabB* and *pabC* were highly abundant, reaching 1,863 and 1,687 counts per million (CPM) in sample 1 and sample 2, respectively (Figure 1A). Genes encoding the enzymes for cobalamin (vitamin B12–827 and 354 CPM), thiamine (vitamin B1, 1,244 and 1.140 CPM) riboflavin (vitamin B2–1,114 and 1,166 CPM), pantothenate (vitamin B5–280 and 415 CPM), biotin (vitamin B7–55 and 75 CPM) and menaquinone (vitamin K – 319 and 239 CPM) (Figure 1A). Genes involved in short-chain fatty acid metabolism were also prevalent. Specifically, *poxB* and *ackA*, encoding enzymes that convert acetyl-CoA to acetate, reached 1,168 and 1,044 CPM in sample 1 and sample 2, respectively (Figure 1A). Additionally, genes associated with propionate biosynthesis were detected in low abundances. Genes involved in quorum sensing pathways, including *luxS*, *agrA*, and *agrC*, were present at 1,363 and 1,381 CPM, and genes encoding bile salt hydrolases, which facilitate bacterial survival in the gastrointestinal tract, reached 309 and 344 CPM. Interestingly, *gadB* and *gadC* genes, involved in the synthesis and transport of neurotransmitter gamma-aminobutyric acid (GABA), were found in 165 and 267 CPM in the two olive samples (Figure 1A).

Using the metagenome dataset, we were able to reconstruct 12 non-redundant MAGs, each with at least 50% completeness and less than 10% contamination. Of these, 10 met the criteria for high-quality MAGs, displaying ≥90% completeness and <5% contamination. Eight MAGs were classified within the *Lactobacillaceae* family, while four did not belong to LAB, namely unclassified *Celerinatantimonas* species, *Sedimentibacterium* sp. 902,168,225, unclassified *Afipia* species, and unclassified species of the JAFKFH01 genus from the *Ferrovibrionaceae* family (Figure 1B). Interestingly, the most abundant MAG corresponded to *Pediococcus parvulus* (63%), followed by *Secundilactobacillus collinoides* (15%), *Lactiplantibacillus pentosus* (8%), *Lactiplantibacillus plantarum* (3%), *Pediococcus ethanolidurans* (3%), *Levilactobacillus suantsaii* (2%), and two additional MAGs identified as an unclassified *Levilactobacillus* and *Lentilactobacillus rapi* (each <1%). The remaining non-LAB MAGs represented less than 1% of RPKM each. Complete biosynthetic modules for vitamin and acetate production were present in the recovered MAGs. Genes encoding for vitamin B12 biosynthesis were found in *L. rapi*, *S. collinoides*, and members of *Celerinatantimonas* and the JAFKFH01 clade. Genetic capacity for vitamin B7 biosynthesis was observed only in *Celerinatantimonas*, whereas vitamin B2 biosynthesis pathways were identified in *L. plantarum*, *Levilactobacillus suantsaii*, *S. collinoides*, and *Celerinatantimonas*. Vitamin B9 biosynthesis module was present in highly abundant *P. parvulus* as well as *S. collinoides, P. ethanolidurans, Sediminibacterium,* and *Celerinatantimonas*. Genes encoding vitamins B1 and B5 biosynthesis were widespread across MAGs, except for certain *Pediococcus, Sediminibacterium*, and *Afipia*. In contrast, menaquinone (vitamin K2) biosynthesis was detected only in *Sediminibacterium*. Acetate production pathways were broadly distributed among LAB and *Celerinatantimonas*.

### 3.2 Impact of storage conditions on total bacterial and LAB abundance and diversity

**Figure 2:**
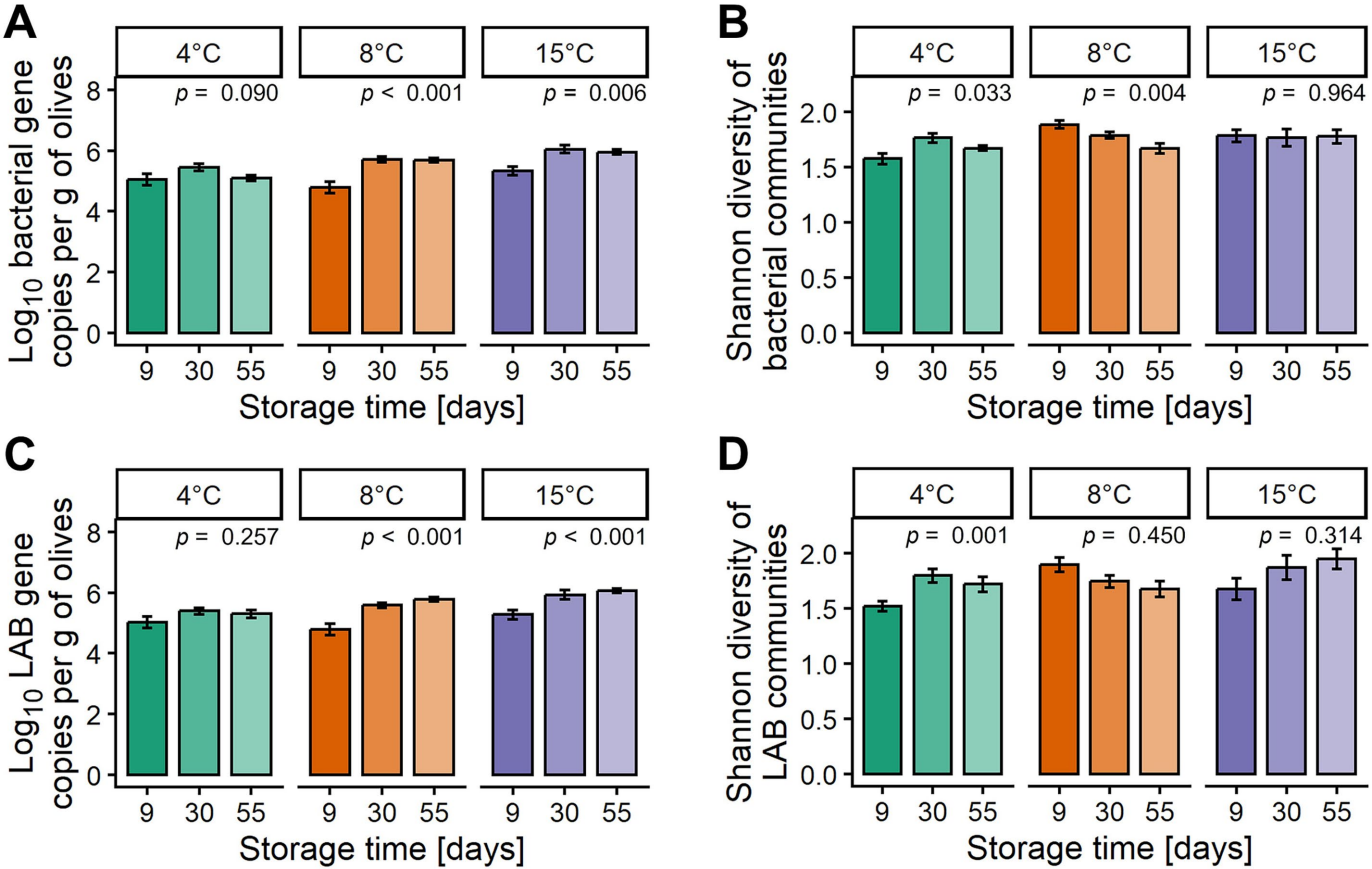
Comparison of absolute bacterial and LAB abundance and Shannon diversity in olive samples that were stored at different temperatures across different storage durations. The bar plots illustrate the variability in total bacterial and LAB abundance **(A,C)** as well as diversity **(B,D)**. Total bacterial **(A)** and LAB **(C)** abundances were measured using a qPCR-based method and subsequently log-transformed. To compare the alpha diversity of total bacterial **(B)** and LAB **(D)** communities, the Shannon index was calculated for each sample type. The green, orange and purple colors represent samples stored at $°C, 8 °C and 15 °C, respectively.

Bacterial absolute abundances varied notably with storage time and temperature. Bacterial abundances were lowest 9 days after storage with 1.1 × 10^5^ gene copy numbers per g (gcn). The highest bacterial abundance was observed at 30 days after storage, with 5.4 × 10^5^ gcn, followed by a slight decrease at 55 days, measuring 3.5 × 10^5^ gcn. Notably, an increase in bacterial abundance was observed with increasing storage temperatures (1.5 × 10^5^, 2.5 × 10^5^, and 6.0 × 10^5^ gcn at 4 °C, 8 °C, and 15 °C, respectively). According to ANOVA, storage time had a significant effect on bacterial abundances, as measured by partial eta-squared (*η*_p_2 = 0.619, *p* < 0.001), as did storage temperature (*η*_p_2 = 0.586, *p* < 0.001). The effect of storage duration differed with temperature and was largest at 8 °C (*η*_p_2 = 0.669, *p* < 0.001), followed by 15 °C (*η*_p_2 = 0.500, *p* = 0.006), and was, interestingly, below the significance threshold at 4 °C (*η*_p_2 = 0.275, *p* = 0.090; Figure 2A). Our results indicate that storage temperature had a greater influence on bacterial absolute abundance than packaging conditions. Higher storage temperatures were associated with more pronounced changes in bacterial absolute abundance, whereas the effect of packaging was comparatively limited (*η*_p_2 = 0.120, *p* = 0.033). Samples stored in glass jars exhibited lower absolute bacterial abundance (2.3 × 10^5^ gcn) than vacuum bags (3.4 × 10^5^ gcn), although the effect depended significantly on time (*η*_p_2 = 0.282, *p* = 0.003) while the interaction with the temperature was suggestive but not significant (*η*_p_2 = 0.134, *p* = 0.075). For example, at 55 days of storage, a higher bacterial absolute abundance was observed in olives stored in glass jars (4.4 × 10^5^ gcn) compared to those stored in vacuum bags (3.2 × 10^5^ gcn), whereas it was lower at 9 (glass: 6.2 × 104 gcn, vacuum: 2.1 × 10^5^ gcn) and 30 days (glass: 4.7 × 10^5^ gcn, vacuum: 6.2 × 10^5^ gcn).

Similar results were obtained when qPCR was performed using the LAB-specific primer pair (Figure 2C). LAB absolute abundances were largely affected by storage time (*η*_p_2 = 0.577, *p* < 0.001) and temperature (*η*_p_2 = 0.450, *p* < 0.001). LAB absolute abundances increased over time when the samples were stored at 8 °C (*η*_p_2 = 0.724, *p* < 0.001) and 15 °C (*η*_p_2 = 0.630, *p* < 0.001), but the changes were not significant at 4 °C (*η*_p_2 = 0.166, *p* = 0.257) (Figure 2C). Similarly to the bacterial result, packaging had a relatively minimal impact on LAB absolute abundance (*η*_p_2 = 0.104, *p* = 0.048). Samples stored in glass jars exhibited lower LAB absolute abundance (2.4 × 10^5^ gcn) than vacuum bags (3.4 × 10^5^ gcn), with the effect depending significantly on time (*η*_p_2 = 0.218, *p* = 0.012). We observed a higher LAB absolute abundance in olives stored in glass jars after 55 days of incubation (5.7 × 10^5^ gcn) compared to those stored in vacuum bags (4.6 × 10^5^ gcn), whereas it was lower at 9 (glass: 4.8 × 10^5^ gcn, vacuum: 5.3 × 10^5^ gcn) and 30 days (glass: 5.6 × 10^5^ gcn, vacuum: 5.7 × 10^5^ gcn). The LAB absolute abundance pattern closely resembles that of the overall bacterial community, indicating that LAB dominate the bacterial populations.

Bacterial alpha diversity in olives, measured by the Shannon index (h′), varied with storage temperature and time (Figure 2B). At 4 °C, diversity was lower in samples stored for 9 days (h′ = 1.57) compared with those stored for 30 days (h′ = 1.76) and 55 days (h′ = 1.67). At 8 °C, diversity declined over time (h′ = 1.88, 1.79, and 1.67 at 9, 30, and 55 days, respectively). In contrast, diversity remained stable throughout the storage period at 15 °C (Figure 2B). Statistical analysis confirmed that storage temperature was the primary factor influencing bacterial diversity (*η*_p_2 = 0.242, *p* = 0.007), with a significant interaction with storage time (*η*_p_2 = 0.332, *p* = 0.005). Neither storage time alone (*η*_p_2 = 0.079, *p* = 0.228) nor packaging (*η*_p_2 < 0.001, *p* = 0.937) impacted bacterial loads significantly.

Incubation time (*η*_p_2 = 0.055, *p* = 0.439), temperature (*η*_p_2 = 0.104, *p* = 0.205), or packaging (*η*_p_2 = 0.005, *p* = 0.715) did not have a significant impact on LAB alpha diversity. However, LAB diversity varied significantly over time during storage at 4 °C (*p* = 0.001). Similar to the total bacterial diversity, a lower LAB diversity was observed after 9 days of storage (h′ = 1.27), compared to olives stored for 30 days (h′ = 1.58), and 55 days (h′ = 1.43) (Figure 2D). Compared to total bacteria, the LAB diversity at 8 °C did not show significant variation over time. Additionally, at 15 °C, the diversity remained stable for both total bacteria and LAB. Furthermore, we observed a significant positive correlation between Shannon diversity of LAB and the total bacterial community across all temperatures, with stronger associations at 4 °C (*R*^2^ = 0.678, *p* < 0.001) and 15 °C (*R*^2^ = 0.650, *p* < 0.001) compared to 8 °C (*R*^2^ = 0.347, *p* = 0.016). These findings suggest that the LAB taxa contributed substantially to overall changes in bacterial diversity, particularly at 4 °C and 15 °C.

### 3.3 The impact of temperature on bacterial community structure and the olive microbial community

**Figure 3:**
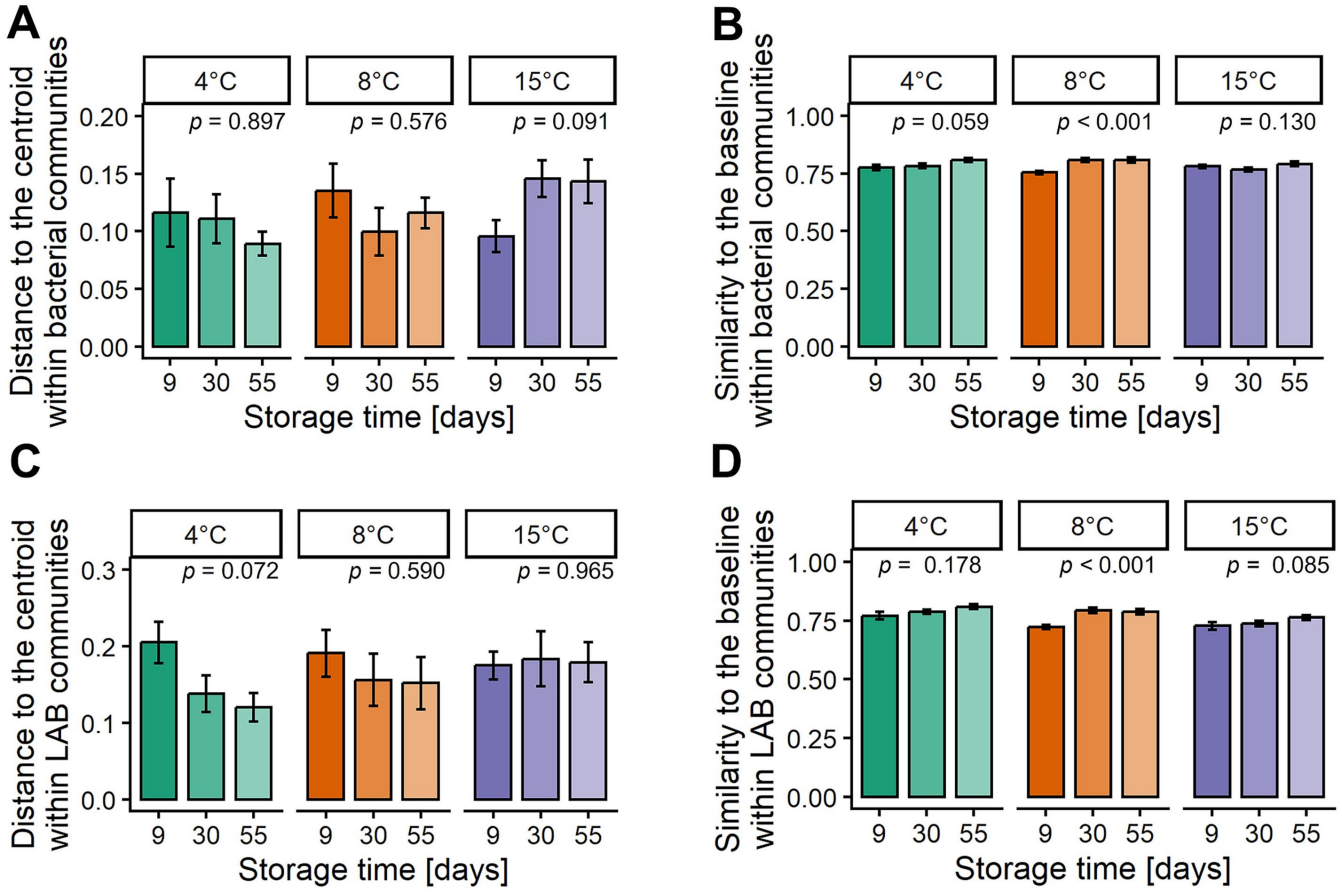
Variability in total bacterial and LAB community composition of olives stored at different temperatures across different storage durations. Bar plots show the distance to the group centroid (beta dispersion) for total bacteria **(A)** and LAB **(C)**, and similarity to the baseline based on total bacteria **(B)** and LAB community compositions **(D)**. *p*-values were calculated using ANOVA. The green, orange and purple colors represent samples stored at $°C, 8 °C and 15 °C, respectively.

To understand how storage conditions influence the overall communities, we performed PERMANOVA on Bray–Curtis dissimilarity matrices. Bacterial beta-diversity was affected by incubation time (*R*^2^ = 9.0%, *p* = 0.004), temperature (*R*^2^ = 9.5%, *p* = 0.006) and packaging (*R*^2^ = 5.1%, *p* = 0.020). To assess microbial stability, defined as the ability of the bacterial community to maintain its composition in response to the tested factors, we analyzed beta dispersion (distance to centroid) across storage time, temperature, and packaging conditions, relative to the baseline community. According to ANOVA, storage time (*η*_p_2 = 0.007, *p* = 0.881), temperature (*η*_p_2 = 0.069, *p* = 0.278), or packaging (*η*_p_2 = 0.053, *p* = 0.164) did not have a significant effect on beta-dispersion of total bacterial communities (Figure 3A). To explore whether the storage led to convergence or divergence from the baseline, we calculated the similarity to the baseline samples as a complement of Bray–Curtis dissimilarity. The stored samples exhibited a high degree of similarity to the baseline (79%), indicating considerable stability in bacterial community composition following storage. Storage time had a significant impact on similarity to the baseline (*η*_p_2 = 0.075, *p* < 0.001), with no significant effect of temperature (*η*_p_2 = 0.011, *p* = 0.340) or packaging (*η*_p_2 = 0.005, *p* = 0.322). Interestingly, the bacterial similarity to the baseline tended to increase with time at 4 °C and 8 °C (Figure 3B), but this trend was not significant.

In LAB, temperature was the strongest driver of LAB community composition (*R*^2^ = 9.8%, *p* = 0.016), followed by packaging (*R*^2^ = 6.2%, *p* = 0.018). Storage time did not have a significant effect on LAB community composition (*R*^2^ = 5.8%, *p* = 0.149). Similar to total bacteria, beta dispersion did not differ significantly with storage time (*η*_p_2 = 0.045, *p* = 0.359), temperature (*η*_p_2 = 0.017, *p* = 0.674) or packaging (*η*_p_2 = 0.004, *p* = 0.723) in LAB (Figure 3C). LAB community similarity to baseline was affected by storage time (*η*_p_2 = 0.063, *p* < 0.001) and temperature (*η*_p_2 = 0.056, *p* < 0.001), but not packaging (*η*_p_2 = 0.002, *p* = 0.392). The effect of time was significant at 8 °C (*η*_p_2 = 0.187, *p* < 0.001), but not at 4 °C (*η*_p_2 = 0.028, *p* = 0.178) or 15 °C (*η*_p_2 = 0.039, *p* = 0.085; Figure 3D). Similar to the overall bacterial community composition, the LAB community composition was relatively stable and remained close to baseline (Figure 3D). Interestingly, the similarity of the LAB community to the baseline was lowest at the shortest storage time across all three temperatures, and the communities generally became more similar to the baseline over time, a trend that was significant at 8 °C (Figure 3D).

### 3.4 Lactic acid bacteria were enriched in olives stored at 15 °C, whereas non-LAB taxa proliferated more at lower temperatures

**Figure 4:**
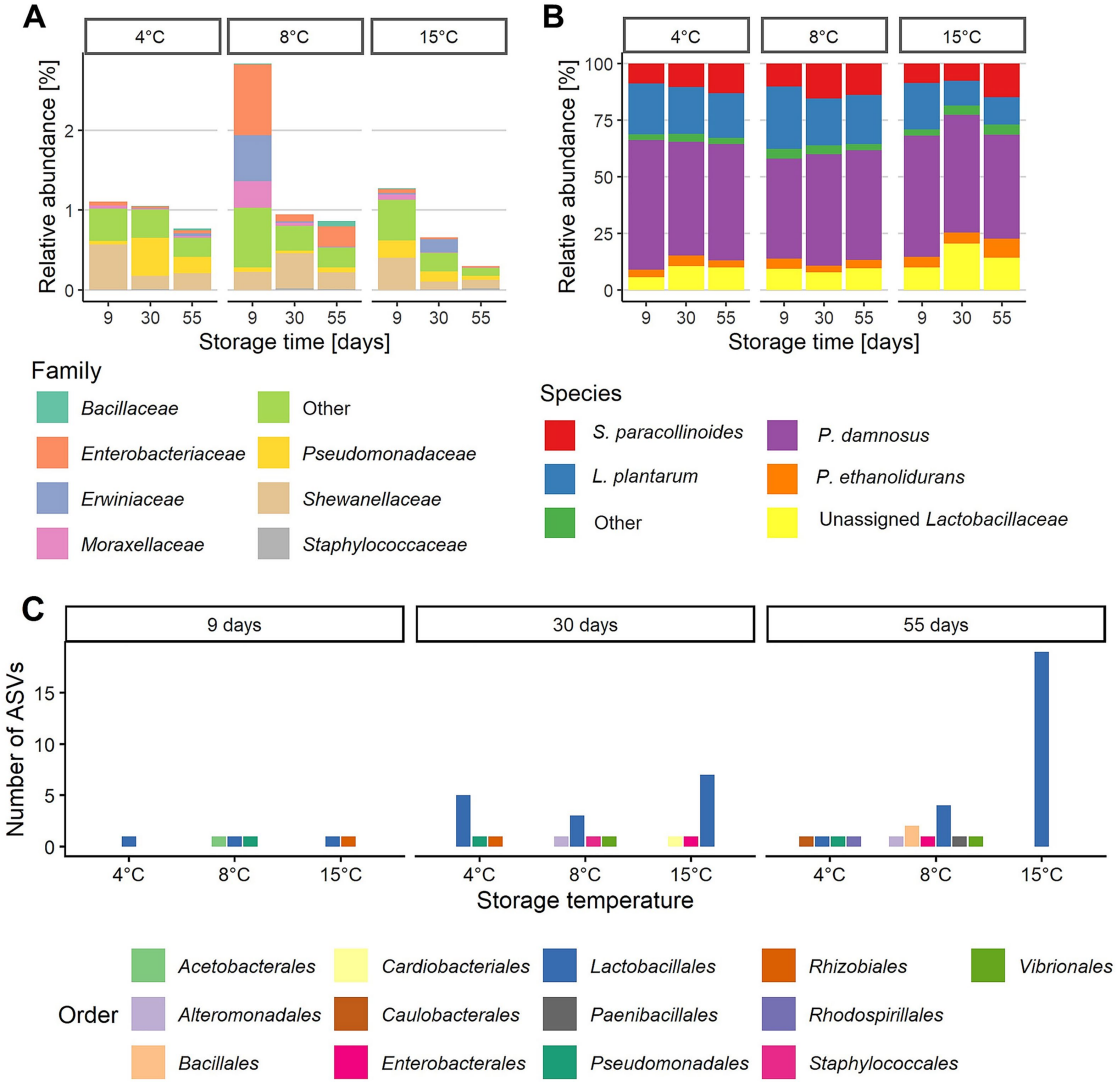
Changes in the bacterial community composition and taxon-specific responses to storage conditions. The bacterial community composition on a family level, excluding the *Lactobacillaceae* family **(A)**, and the LAB composition at species level **(B)** across different storage conditions. The number of differentially abundant ASVs, enriched at different storage temperatures across different storage durations, annotated at the order level **(C)**.

The relative abundance of LAB increased with storage time. Relative abundance of *Lactobacillaceae* increased from 94.6% to 98.2% of total bacterial reads 9 days after storage. This pattern persisted across all time points and conditions (Figure 4A). When examining the species level, the LAB communities resembled the baseline as they were dominated by four highly abundant species, namely *Pediococcus damnosus* (51%), *Lactiplantibacillus plantarum* (19%), *Secundilactobacillus paracollinoides* (12%), and *Pediococcus ethanolidurans* (4%), in similar abundance at all storage conditions (4B). LEfSE analysis supported strong enrichment of LAB at higher storage temperatures over time a total of 48 unique ASVs were significantly enriched at a certain temperature, at least at one storage time point. Of these, 69% belonged to LAB of the family *Lactobacillaceae*, among them 8 *Pediococcus*, 7 *Lactiplantibacillus*, 6 *Lentilactibacillus* and 5 *Levilactobacillus* ASVs (Supplementary Table S1). *Pediococcus, Lentilactobacillus* and *Levilactobacillus* ASVs were enriched at 15 °C, whereas *Lactiplantibacillus* was enriched at 4 °C and 8 °C (Supplementary Table S1). Only 6 ASVs were differentially abundant at specific temperature at 9 days of storage (Figure 4C). At 30 days of storage, 22 ASVs appeared differentially abundant, with the highest number of LAB enriched at 15 °C (9 ASVs) compared to 4 °C and 8 °C (7 and 6 ASVs, respectively). This pattern was even stronger after 55 days; the majority of differentially abundant LAB ASVs were enriched at 15 °C (Figure 4C). These findings indicated that LAB were more prevalent in olives stored at higher temperatures. In contrast, non-LAB, belonging to *Gamma-proteobacteria*, *Alpha-proteobacteria* and *Bacilli* were sporadically enriched mainly at 4 °C and 8 °C (Figure 4C). Notably, among the taxa enriched at these temperatures were genera potentially relevant to food safety, including *Pseudomonas*, *Vibrio*, *Staphylococcus*, and *Salmonella* (Supplementary Table S1).

## 4 Discussion

This study reveals novel insights into the beneficial health traits of the microbiota in Kalamata olives. We showed that the olive microbiota harbors genes involved in the biosynthesis of several essential compounds, including B-complex vitamins, short-chain fatty acids (SCFAs), and GABA. These predicted functions suggest that the olive microbiome may contribute not only to product preservation but also to characteristics of the final product that could influence its nutritional and bioactive composition.

The olive microbiome is characterized by a diverse community of LAB and reflects the well-established role of these organisms in olive fermentation and post-fermentation stability. The surprising fact in Kalamata olives was the high diversity within the *Lactobacillaceae* family as well as the occurrence of other microorganisms belonging to many different families, e.g., *Bacillaceae, Staphylococcaceae, Enterobacteriaceae, Pseudomonadaceae*, and *Shewanellaceae*. This community structure is in agreement with previous studies of Kalamata olives as well as olives from different varieties, which reported dominance by *Lactiplantibacillus, Pediococcus* or oher *Lactobacillaceae* accompanied by *Gamma-proteobacteria* and other bacteria in variable but generally low relative abundances (Kazou et al., 2020; Michailidou et al., 2021; Kamilari et al., 2023; Soto-Giron et al., 2021). Another important finding was the high abundance of genes for the biosynthesis of multiple vitamins within the B complex group, vitamin K, SCFAs and GABA in olive-associated bacteria. In addition, genes associated with bacterial colonization in the human digestive system, namely genes encoding bile salt hydrolases and quorum-sensing systems, were present in the olive microbiome as well. Their presence suggests that some olive-associated bacteria may survive in the gastrointestinal tract and colonize it transiently, although this would need to be confirmed by functional assays and experimental validation. Comparable functional potentials were observed in microorganisms associated with other fermented food environments, such as kimchi (Chun et al., 2017), tempeh (Wicaksono et al., 2024) and green olives (Soto-Giron et al., 2021), which, together with this study, highlight the importance of the food microbiome functional potential for human health.

The most abundant species based on MAGs was *Pediococcus parvulus,* which was likely assigned to the closely related *Pediococcus damnosus* in the amplicon dataset due to the limited resolution of the 16S rRNA gene*. Pediococcus* species are often found in olives and were reported in variable, sometimes high abundances in Kalamata olives as well (Michailidou et al., 2021; Kazou et al., 2020). In this study, *P. parvulus* MAG contained the biosynthetic pathways for the production of vitamins B5 and B9, as well as acetate, highlighting its potential functional importance in Kalamata olive fermentation. *Pediococcus parvulus*, commonly used in oat fermentation, has previously been shown to inhibit food spoilage microorganisms (Immerstrand et al., 2010), suggesting that this species may contribute to both product stability and functional capacity in different types of foods. Other olive-associated LAB MAGs also encoded pathways for the biosynthesis of several B-complex vitamins. For instance, the second most abundant MAG, *Secundilactobacillus collinoides* (likely assigned as *Secundilactobacillus paracollinoides* in the amplicon dataset), carried the genes for the production of all vitamins detected in LAB in this study. Notably, all recovered MAGs identified as LABs contained the genes for the biosynthesis of acetate, one of the key SCFAs, which can facilitate microbial stability, sensory development (Basso et al., 2021) as well as health promotion (Moffett et al., 2020). Taken together, these findings suggest that LAB, due to both their abundance and putative rich metabolic repertoire, are likely the main contributors to the functional and nutritional potential of the Kalamata olive microbiome. Notably, our study assessed the functional potential solely at the baseline; however, the storage-derived changes could be inferred from the shifts in taxonomic composition. Given the consistent presence of the dominant taxa, significant shifts in functional potential are therefore not anticipated.

Moreover, microorganisms belonging to non-LAB taxa also contained genetic potential for beneficial functions. For instance, a MAG belonging to the *Celerinatantimonas* genus possesses the complete set of genes necessary for the biosynthesis of all assessed B complex vitamins, including the exclusive presence of the vitamin B7 biosynthesis pathway, as well as acetate. Interestingly, *Celerinatantimonas* spp. are halophilic bacteria that have been associated with the formation of gas-pocket defects in Spanish-style green olives through the production of gaseous metabolites during storage and processing (de Castro et al., 2022). Similarly, a MAG belonging to *Sediminibacterium* MAG harbors genes related to vitamin K production. These findings indicate that putative health-promotion capabilities are not restricted to LAB, underscoring the role of non-LAB taxa from the olives in influencing product quality. Although speculative, some of these non-LAB taxa may warrant further investigation due to their functional potential, reflecting the growing interest in characterizing non-LAB microorganisms with beneficial effects for human health (Lee et al., 2024). However, any potential application of these microorganisms would require careful evaluation of their safety and suitability.

Although storage and packaging modulated microbial abundance and diversity, the overall functional potential of the community likely remained stable across tested conditions. The modest effect of packaging type on community structure suggests that functional resilience is largely maintained regardless of container material. Storage temperature and duration primarily influenced bacterial abundance and community composition, but did not result in the removal of any key LAB species. Higher temperatures favored LAB proliferation and possibly greater expression of metabolic functions such as SCFAs and vitamin biosynthesis. In contrast, lower temperatures promoted the persistence of psychrotrophic and oxidative taxa, potentially lowering the community’s potential to produce health-beneficial compounds. A possible explanation for this pattern is the oxygen sensitivity of LAB (Feng and Wang, 2020), which grew better at higher temperatures, where higher metabolic rates led to faster oxygen depletion from closed containers. Interestingly, storage at 4 °C limited LAB proliferation and favored aerobic or psychrotrophic taxa, including members of *Pseudomonadales*, *Acinetobacter*, *Salmonella, Vibrio* and *Staphylococcus*. The detection of such low-abundant taxa highlights the complexity of olive microbiomes beyond LAB. It is important to note that these groups include species associated with opportunistic and food-borne pathogens (Skowron et al., 2022). However, the low relative abundance and the limitations of molecular methods deployed in this study limit the conclusions regarding food safety risks. Moreover, these organisms are common in table olives (Lucena-Padrós et al., 2015; López-García et al., 2021; Mougiou et al., 2023) and reports linking table olives to outbreaks of the mentioned taxa appear to be rare. This could, at least partially, be explained by the suppressive nature of the fermented olives, which can often effectively suppress foodborne pathogens (Romero-Gil et al., 2018; Grounta et al., 2013). Overall, although temperature significantly influenced bacterial community composition, the continued presence of dominant LAB species across conditions indicates that the functional potential of the microbiota was largely conserved. Notably, storage at 15 °C – broadly resembling traditional cellar or basement conditions—supported the most rapid LAB proliferation, suggesting that moderate temperatures may optimize fermentative activity and product stability. However, such conditions might significantly reduce the abundance of potentially beneficial non-LAB taxa, underscoring the need for further targeted investigations before specific storage recommendations can be made.

Despite providing a comprehensive overview of bacterial dynamic changes during storage, this study has several limitations. First, the work focused on a single olive type, which may limit the generalizability of the observed microbial patterns to other cultivars or fermentation styles. Second, fungal communities were not included in the analysis, although yeasts have been reported to be an important component of the Kalamata olive microbiome (Michailidou et al., 2021). Furthermore, the functional potential is inferred from the metagenomic sequencing of the two baseline samples. The low sample size, as well as the lack of biochemical validation, limit the interpretation. For future work, it would be highly beneficial to study the actual correlations between specific microbial taxa and concentrations of bioactive metabolites. Finally, important biochemical parameters for storage and fermentation, such as brine concentration, pH, and storage/fermentation time before the experiment, were unavailable, which limits the interpretation of the results.

Overall, our findings indicate that the bacterial communities in olives form a metabolically rich and resilient ecosystem and potentially play a key role in product value. Moreover, our study demonstrates that typical storage practices influence the overall bacterial community composition. Specifically, we observed that LAB proliferation is more prevalent at higher temperatures, whereas lower temperatures may favor the enrichment of non-LAB taxa, yet the dominant LAB species carrying important functions remain stable. Future research could expand upon these results by incorporating sensory analysis to elucidate the relationship between microbial community dynamics, functional potential, and product quality.

## Data Availability

The datasets presented in this study can be found in online repositories. The names of the repository/repositories and accession number(s) can be found in the article/Supplementary material.

## Footnotes

1
https://github.com/wwood/CoverM
