# Supplementary material for: Metagenomic analysis reveals functional potential and storage-driven dynamics of the Kalamata olive microbiome
Source: Front Microbiol. 2026 Jul 8;17:1890405. doi: 10.3389/fmicb.2026.1890405 (PMC13388459; doi:10.3389/fmicb.2026.1890405)
Supplement: Supplementary file 1 [file Table_1.docx]

Supplementary Material

**Supplementary table:** ASVs enriched at specific storage durations and temperatures annotated at the genus level

| Enriched at | Storage time | ef_lda | p-value (adjusted) | Genus |
| --- | --- | --- | --- | --- |
| 15°C | 9 days | 2.899812 | 0.013177 | *Loigolactobacillus* |
| 4°C | 9 days | 2.636597 | 0.049105 | *Lactiplantibacillus* |
| 8°C | 9 days | 2.810626 | 0.022266 | *Acetobacter* |
| 4°C | 9 days | 4.882742 | 0.096972 | *Pediococcus* |
| 8°C | 9 days | 3.836002 | 0.057963 | Unclassified *Lactobacillaceae* |
| 8°C | 9 days | 3.213651 | 0.080511 | *Acinetobacter* |
| 15°C | 30 days | 4.729361 | 0.003625 | Unclassified *Lactobacillaceae* |
| 8°C | 30 days | 2.149038 | 0.034502 | *Lacticaseibacillus* |
| 15°C | 30 days | 3.350699 | 0.082325 | *Pediococcus* |
| 4°C | 30 days | 3.206803 | 0.010504 | *Lactiplantibacillus* |
| 15°C | 30 days | 3.151541 | 0.077583 | *Pediococcus* |
| 8°C | 30 days | 2.623311 | 0.046959 | *Vibrio* |
| 15°C | 30 days | 2.990316 | 0.028729 | *Secundilactobacillus* |
| 8°C | 30 days | 3.382451 | 0.064397 | *Ferrimonas* |
| 4°C | 30 days | 3.053105 | 0.032896 | *Serratia* |
| 4°C | 30 days | 2.449655 | 0.015552 | *Bradyrhizobium* |
| 8°C | 30 days | 3.68218 | 0.014495 | *Lactiplantibacillus* |
| 8°C | 30 days | 4.539542 | 0.096972 | *Secundilactobacillus* |
| 8°C | 30 days | 2.32392 | 0.008223 | *Staphylococcus* |
| 15°C | 30 days | 2.635052 | 0.082113 | Unclassified *Lactobacillaceae* |
| 15°C | 30 days | 3.690921 | 0.005427 | *Pediococcus* |
| 8°C | 30 days | 3.819124 | 0.028733 | *Secundilactobacillus* |
| 8°C | 30 days | 4.65032 | 0.029413 | *Lactiplantibacillus* |
| 4°C | 30 days | 3.617336 | 0.0035 | *Pseudomonas* |
| 15°C | 30 days | 2.649888 | 0.06225 | *Pediococcus* |
| 15°C | 30 days | 3.014775 | 0.096972 | *Levilactobacillus* |
| 15°C | 30 days | 2.966657 | 0.020627 | *Pantoea* |
| 4°C | 30 days | 3.935965 | 0.017581 | *Lactiplantibacillus* |
| 4°C | 30 days | 2.4989 | 0.005412 | *Enhydrobacter* |
| 15°C | 30 days | 3.396504 | 0.024458 | *Lentilactobacillus* |
| 15°C | 30 days | 3.246101 | 0.082911 | *Lentilactobacillus* |
| 15°C | 30 days | 3.265946 | 0.088477 | *Pediococcus* |
| 4°C | 30 days | 2.260227 | 0.01975 | *Streptococcus* |
| 15°C | 55 days | 2.751475 | 0.062581 | *Lentilactobacillus* |
| 15°C | 55 days | 3.593379 | 0.013873 | Unclassified *Lactobacillaceae* |
| 15°C | 55 days | 2.450925 | 0.033687 | Unclassified *Lactobacillaceae* |
| 15°C | 55 days | 2.76101 | 0.04961 | Unclassified *Lactobacillaceae* |
| 8°C | 55 days | 2.990586 | 0.006605 | *Salmonella* |
| 4°C | 55 days | 2.905628 | 0.036305 | *Loigolactobacillus* |
| 15°C | 55 days | 2.598707 | 0.003074 | *Lentilactobacillus* |
| 15°C | 55 days | 3.888087 | 0.003042 | *Lentilactobacillus* |
| 8°C | 55 days | 3.274893 | 0.07083 | *Lactiplantibacillus* |
| 15°C | 55 days | 2.709448 | 0.042191 | Unclassified *Lactobacillaceae* |
| 15°C | 55 days | 4.487074 | 0.072811 | *Pediococcus* |
| 4°C | 55 days | 2.499715 | 0.000315 | Unclassified *Caulobacteriaceae* |
| 15°C | 55 days | 2.72182 | 0.007784 | *Lentilactobacillus* |
| 15°C | 55 days | 2.899233 | 0.002747 | Unclassified *Lactobacillaceae* |
